# Beta-lactamase resistance genes in *Enterobacteriaceae* from Nigeria

**DOI:** 10.4102/ajlm.v11i1.1371

**Published:** 2022-02-22

**Authors:** Babafela B. Awosile, Michael Agbaje, Oluwawemimo Adebowale, Olugbenga Kehinde, Ezekiel Omoshaba

**Affiliations:** 1 Texas Tech University School of Veterinary Medicine, Amarillo, Texas, United States; 2 Department of Veterinary Microbiology, College of Veterinary Medicine, Federal University of Agriculture, Abeokuta, Nigeria; 3 Department of Veterinary Public Health and Preventive Medicine, College of Veterinary Medicine, Federal University of Agriculture, Abeokuta, Nigeria

**Keywords:** antimicrobial resistance, beta-lactamase gene, Nigeria, review, epidemiology

## Abstract

**Background:**

Beta-lactamase genes are one of the most important groups of antimicrobial resistance genes in human and animal health. Therefore, continuous surveillance of this group of resistance genes is needed for a better understanding of the local epidemiology within a country and global dissemination.

**Aim:**

This review was carried out to identify different beta-lactamase resistance genes reported in published literature from Nigeria.

**Methods:**

Systematic review and meta-analysis was carried out on eligible Nigerian articles retrieved from electronic literature searches of PubMed^®^, African Journals Online, and Google Scholar published between January 1990 and December 2019. The Preferred Reporting Items for Systematic Reviews and Meta-Analyses method was adopted to facilitate clarity and transparency in reporting review findings.

**Results:**

Fifty-seven articles were included. All beta-lactamases reported were detected from Gram-negative bacteria, particularly from *Enterobacteriaceae*. Thirty-six different beta-lactamase genes were reported in Nigeria. These genes belong to the narrow-spectrum, AmpC, extended-spectrum and carbapenemase beta-lactamase resistance genes. The pooled proportion estimate of extended-spectrum beta-lactamase genes in Nigeria was 31% (95% confidence interval [CI]: 26% – 36%, *p* < 0.0001), while the estimate of the *bla*_CTX-M-15_ gene in Nigeria was 46% (95% CI: 36% – 57%, *p* < 0.0001). The proportion estimate of AmpC genes was 32% (95% CI: 11% – 52%, *p* < 0.001), while the estimate for carbapenemases was 8% (95% CI: 5% – 12%, *p* < 0.001).

**Conclusion:**

This study provides information on beta-lactamase distribution in Nigeria. This is necessary for a better understanding of molecular epidemiology of clinically important beta-lactamases, especially the extended-spectrum beta-lactamases and carbapenemases in Nigeria.

## Introduction

Beta-lactam antimicrobials are one of the most important groups of antimicrobial drugs used in both human and animal health. Antimicrobials such as extended-spectrum cephalosporins and carbapenems have been categorised by the World Health Organization as ‘last resort’ and ‘critically important antimicrobials’ because antimicrobial alternatives for treating last resort antimicrobial resistant bacteria is limited.^1^ However, resistance to last resort antimicrobials is occurring rapidly on a global scale.^2^ Most resistance to beta-lactams in *Enterobacteriaceae* is mainly due to the production of beta-lactamases, which are often encoded either on the chromosome or the plasmid.^3,4^ The production of beta-lactamases such as extended-spectrum beta-lactamases (ESBLs), AmpC beta-lactamases, and carbapenemase beta-lactamases have increasingly been detected globally in food and companion animals, wildlife, humans, and the environment.^4,5^ The dissemination of several beta-lactamase genes across different resistant bacterial populations from different hosts and environments illustrates that antimicrobial resistance (AMR) is a One Health challenge.^6^

Beta-lactamase production in *Enterobacteriaceae* is a public health concern due to the possibility of therapeutic failure, serious consequences for infection control and increased risk of morbidity and mortality in animals and humans.^7^ The predominant ESBL genes encountered are *bla*_CTX-M_, *bla*_TEM_, and *bla*_SHV_. The prevalent AmpC beta-lactamase is *bla*_CMY-2_, while for carbapenemases, *bla*_OX__A-48_ and *bla*_NDM-1_ have been reported globally.^5^ Although beta-lactamase genes are globally disseminated, they are not equally prevalent among human and animal bacteria. Also, the occurrence and prevalence of these resistance genes varies across different geographic regions. For instance, while *bla*_CTX-M__-15_ is widely disseminated and has been reported in almost every region of the world, AmpC *bla*_CMY-2_ has been mostly encountered in North America in both animal and human hosts.^8^

Therefore, there is a need for continuous surveillance of beta-lactamase resistance genes to better understand the local and global epidemiology of these genes. While detailed national AMR information exists for high-income European and North American countries through integrative surveillance, this is often lacking in most low- and middle-income African countries including Nigeria. Nigeria’s AMR surveillance is in its infancy; thus, the generation of AMR data through the systematic review of published literature is still a useful tool that can give a glimpse of the AMR situation in Nigeria. This systematic review was carried out to identify the different beta-lactamase resistance genes reported in published Nigerian literature, to describe the distribution of these genes between animal, human and environmental settings, and to estimate the proportion of the different beta-lactamase resistance genes in Nigeria. This systematic review was conducted per the Preferred Reporting Items for Systematic Reviews and Meta-Analyses checklist.^9^

## Methods

### Literature search and data sources

The literature search was conducted in PubMed^®^, Google Scholar and African Journals Online (AJOL) electronic databases using a combination of Boolean operators (AND, OR) and predefined keywords. We used the following terms for our search: beta-lactamases AND Nigeria OR beta-lactamase resistance genes AND Nigeria, *bla*_CTX-M_ AND Nigeria, *bla*_TEM_ AND Nigeria, *bla*_SHV_ AND Nigeria, *bla*_OXA_ AND Nigeria, carbapenemases AND Nigeria, AmpC beta-lactamase resistance AND Nigeria, and ESBL resistance AND Nigeria. The search was limited to publications between January 1990 to December 2019. The primary aim of the review was to determine the distribution and different types of beta-lactamase genes circulating in Nigeria across the health sectors. Title screenings of the articles were done using the following eligibility criteria: (1) the study location must be Nigeria and (2) the study must have reported the detection of any type of beta-lactamase genes. Afterwards, an abstract review was done to determine the relevance of each article to the review’s objectives and purpose. For an article to be included it must report the phenotypic antimicrobial susceptibility testing method used and the molecular techniques used to detect beta-lactamase resistance genes. Furthermore, a supplementary literature search was done by reviewing the references of eligible articles. Studies that reported beta-lactamase production based on phenotypic synergy test without molecular beta-lactamase gene detection were excluded from the review. Included articles were then sorted into qualitative and quantitative categories. All articles that reported the molecular detection of at least one beta-lactamase resistance gene were included in the qualitative, while studies with extractable data on the proportions of different types of beta-lactamase resistance genes from animals, humans and the environment were further considered for quantitative meta-analysis.

### Data extraction and analysis

The data were extracted into a Microsoft Office Excel 2010 spreadsheet (Microsoft Corporation, Redmond, Washington, United States). For each eligible study, data extracted included: first author’s details, publication year, sample type (animal faeces, retail meat products, human clinical samples, environmental samples), sample source (animal, human or environment) and study location or geopolitical zone. Also, the number and type of bacteria isolated, the beta-lactamase gene detected, the number of isolates phenotypically positive for the beta-lactamase production and the number of bacterial isolates genotypically positive for beta-lactamase genes were recorded. The antimicrobial susceptibility testing method (disc diffusion, broth microdilution, agar dilution, E-test or automated methods) and the beta-lactamase genotyping and phenotyping methods were noted. The proportion (with 95% confidence interval [CI]) of each beta-lactamase gene as reported for each study was calculated by dividing the number of bacteria positive for the beta-lactamase gene by the total number of bacteria positive phenotypically or the total number of bacteria isolated depending on the data available.

Random effects meta-analysis was used to calculate the pooled (weighted) proportions for the different types of the beta-lactamase groups with 95% CIs. The analysis was done to allow for any heterogeneity between studies. Studies reporting a low number of bacterial isolates (< 10 isolates) were not included in the meta-analysis. The pooled prevalence and each study’s estimates were presented using forest plot. The *I*^2^ statistic (a measure of inconsistency) was used to assess the variation between studies due to heterogeneity. A value of 0% means there was no observed heterogeneity; increasing values indicate increasing heterogeneity. The *I*^2^ statistic with values 25% or less were subjectively considered as low, 26% – 50% as moderate, and higher than 50% as substantial heterogeneity. Subgroup analysis was performed to account for potential sources of heterogeneity between studies. A separate meta-analysis was carried out for each of the dominant beta-lactamase groups. Statistical significance (*p*) was set at 0.05 while statistical analysis was carried out using STATA SE version 15.0 (College Station, Texas, United States).

### Ethical considerations

This article followed all ethical standards for research without direct contact with human or animal subjects.

## Results

### Study characteristics

**FIGURE 1:**
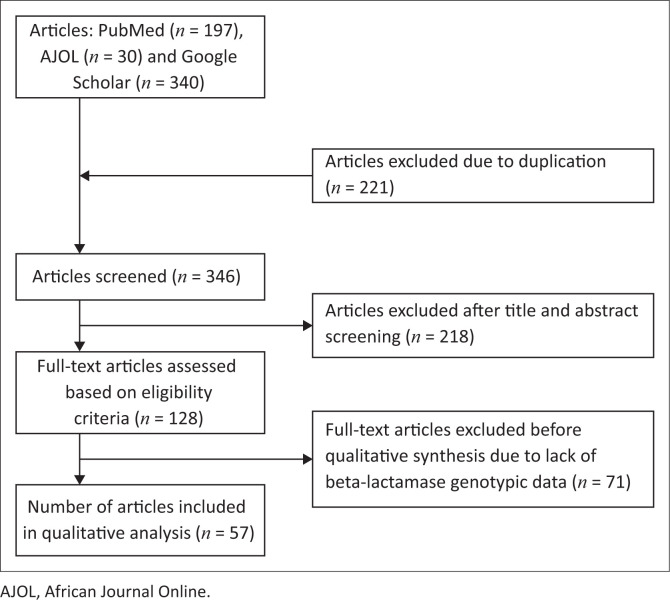
Flow diagram summarising the process of literature search and selection.

Systematic search from three electronic databases identified 567 articles (197 from PubMed^®^, 30 from AJOL and 340 from Google Scholar) (Figure 1). A total of 510 articles were excluded based on the selection criteria (Figure 1). Fifty-seven articles were included in the qualitative review, of which 11 were animal, 38 human and 8 environmental studies. For the animal studies, isolates were from poultry, pigs, cattle, pigeons and ducks. However, all the human studies were hospital-based, with the beta-lactamases reported from clinical samples collected within the hospitals in Nigeria. For the environmental studies, samples were collected from the beach, river, wastewater, and sources of drinking water.

The majority of the studies were carried out in South West Nigeria (*n* = 36), while the rest were carried out in the other regions including South East (*n* = 7), North Central (*n* = 6), North East (*n* = 3), North West (*n* = 1) and South South (*n* = 2). Sixteen (*n* = 16) studies used the broth microdilution method to determine the minimum inhibitory concentrations; other methods used for minimum inhibitory concentrations determination included E-test (*n* = 3) and Vitek-2 (*n* = 2). However, the disc diffusion method (*n* = 36) was mainly used for the antimicrobial susceptibility of bacteria. Forty-three of the 57 studies reported the use of phenotypic screening methods for beta-lactamase production; this included a modified Hodge test and Carba test (*n* = 6) for carbapenemases production as well as the double-disc synergy test (*n* = 37) for other beta-lactamase production. Five different genotypic methods including polymerase chain reaction (PCR), gene sequencing, whole genome sequencing (WGS), isoelectric point and restriction fragment length polymorphism (RFLP) were reported by the studies. Forty-four studies used PCR alone (*n* = 44), eight studies used PCR and gene sequencing, two used PCR with an isoelectric point, one used PCR and whole-genome sequencing, one used PCR with RFLP while only one study used whole-genome sequencing alone for the genotypic detection of various beta-lactamase resistance genes. Among the 57 studies reviewed, the majority of beta-lactamases were detected in *Enterobacteriaceae* (*n* = 54), followed by *Acinetobacter baumannii* (*n* = 2) and *Vibrio* spp (*n* = 1).

### One Health distribution of beta-lactamase resistance genes in Nigeria

Thirty-six different beta-lactamase genes were detected and reported in the 57 studies (Table 1). Seventeen genes were detected in animals, 28 in humans, and 12 in the environment. These genes belong to the AmpC,^10,11,12,13,14,15,16,17,18,19,20,21,22,23,24^ extended-spectrum,^10,11,12,13,14,15,17,19,20,23,24,25,26,27,28,29,30,31,32,33,34,35,36,37,38,39,40,41,42,43,44,45,46,47,48,49,50,51,52,53,54,55,56^ narrow-spectrum,^13,14,15,16,19,24,32,35,37,43,57,58,59^ and carbapenemase beta-lactamase resistance genes.^19,22,59,60,61,62,63,64,65,66^ Eight genes (*bla*_CTX-M-1_, *bla*_CTX-M-14_, *bla*_DHA_, *bla*_GES-1_, *bla*_OXA-1_, *bla*_OXA-2,_ *bla*_TEM-1_ and *bla*_VEB-1_) were found in animals and humans; five genes (*bla*_NDM-1_, *bla*_SHV-1,_ *bla*_SHV-2_, *bla*_SHV-11_, and *bla*_SHV-12_) were common to both humans and the environment while none of the genes was unique to both animals and the environment (Table 1). Four genes, namely *bla*_AmpC_, *bla*_CMY_, *bla*_TEM-1_ and the international pandemic *bla*_CTX-M-15_, were reportedly detected from animals, humans and the environment. No carbapenemase gene was reported in animals but seven beta-lactamase genes (*bla*_ACC_, *bla*_ACT-5_, *bla*_CMY-2_, *bla*_CTX-M-27_, *bla*_CTX-M-55_, *bla*_ECB_, and *bla*_FOX-1_) were unique to animals alone, 12 (*bla*_CTX-M-2_, *bla*_KPC_, *bla*_NDM-5_, *bla*_OXA-10_, *bla*_OXA-23_, *bla*_OXA-48_, *bla*_OXA-181_, *bla*_SHV-28_, *bla*_SHV-112_, *bla*_TEM-2_, *bla*_VIM-1_, and *bla*_VIM-2_) were unique to humans, while only two genes (*bla*_VIM-5_ and *bla*_Z_) were unique to the environment.

**TABLE 1:** Distribution of beta-lactamase resistance genes between animals, humans and environmental settings in Nigeria (1900–2019).

Beta-lactamase type	Gene	Animals	Humans	Environment	Reference
AmpC beta-lactamases	*bla*_ampC_	1†	5	1	10,11,12,13,14,15,16,17,18,19,20,21,22,23,24
	*bla*_CMY-2_	1	-	-	
	*bla*_CMY_ like	1	1	1	
	*bla*_ACT-5_	1	-	-	
	*bla*_ACC_	1	-	-	
	*bla*_FOX-1_	2	-	-	
	*bla*_DHA-1_	1	1	-	
	*bla*_ECB_	1	-	-	
Extended-spectrum beta-lactamases	*bla*_ctxm_ like	3	12	1	10,11,12,13,14,15,17,19,20,23,24,25,26,27,28,29,30,31,32,33,34,35,36,37,38,39,40,41,42,43,44,45,46,47,48,49,50,51,52,53,54,55,56
	*bla*_ctxm-1_	2	2	-	
	*bla*_ctxm-2_	-	1	-	
	*bla*_ctxm-14_	1	1	-	
	*bla*_ctxm-15_	6	14	2	
	*bla*_ctxm-27_	1	-	-	
	*bla*_CTX-M-55_	1	-	-	
	*bla*_SHV-2_	-	1	1	
	*bla*_SHV-12_	-	2	1	
	*bla*_SHV-28_	-	1	-	
	*bla*_SHV-112_	-	1	-	
	*bla*_OXA-10_	-	1	-	
	*bla*_VEB-1_	1	1	-	
	*bla*_GES_	1	1	-	
Narrow-spectrum beta-lactamases	*bla*_OXA-1_	2	4	-	13,14,15,16,19,24,32,35,37,43,57,58,59
	*bla*_OXA-2_	1	1	-	
	*bla*_SHV-1_	-	2	1	
	*bla*_SHV-11_	-	2	1	
	*bla*_TEM-1_	3	6	1	
	*bla*_TEM-2_	-	1	-	
	*bla*_Z_	-	-	1	
Carbapenemases	*bla*_KPC_	-	1	-	19,22,59,60,61,62,63,64,65,66
	*bla*_OXA-23_	-	1	-	
	*bla*_OXA-48_	-	1	-	
	*bla*_OXA-181_	-	3	-	
	*bla*_NDM-1_	-	5	1	
	*bla*_NDM-5_	-	1	-	
	*bla*_VIM-1_	-	5	-	
	*bla*_VIM-2_	-	1	-	
	*bla*_VIM-5_	-		1	

Note: The values in each cell represent the number of articles reporting the beta-lactamase genes.

### Proportion estimates of extended-spectrum beta-lactamase genes in Nigeria

**FIGURE 2:**
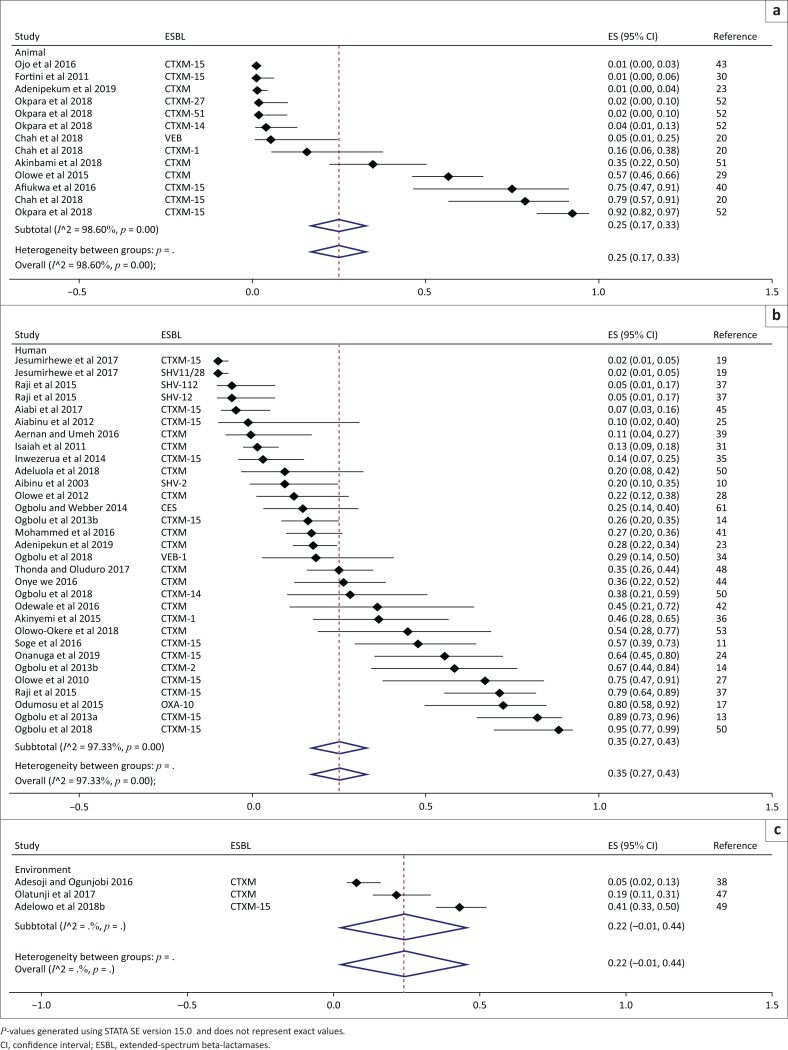
Subgroup analysis and forest plot of proportion estimates of extended-spectrum beta-lactamases for animal (a), human (b) and environmental settings (c) in Nigeria (1990–2019).

**FIGURE 3:**
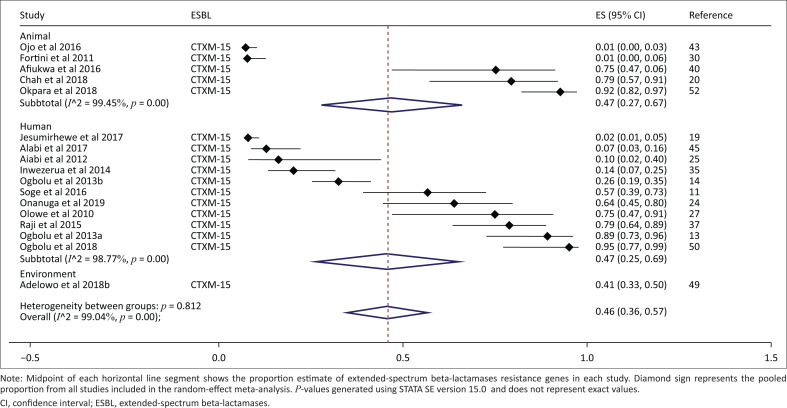
Subgroup analysis and forest plot of proportion estimates of *bla*_CTX-M-15_ extended-spectrum beta-lactamase in Nigeria (1990–2019).

Thirty-two studies were included in the meta-analysis for the generation of the overall pooled estimate of ESBL. The overall pooled proportion of ESBL was 31% (95% CI: 26% – 36%, *p* < 0.001). The overall between-study heterogeneity was significant and substantial (*I*^2^ = 97.87 %, *p* < 0.001). Between the studies, the proportions of ESBL genes range from 1% to 95% (Figure 2). The overall proportion estimate of ESBLs in human-based studies was 35% (95% CI: 27% – 43%, *p* < 0.001), in animal studies was 25% (95% CI: 17% – 33%, *p* < 0.001) and in environmental-based studies was 22% (95% CI: 0% – 44%, *p* = 0.06). A separate meta-analysis was conducted to determine the proportion estimate of *bla*_CTX-M-15_ producing *Enterobacteriaceae* in Nigeria (Figure 3); 17 studies were included in the quantitative analysis. The overall pooled proportion was 46% (95% CI: 36% – 57%), the unexplained between-study heterogeneity was significant and substantial (*I*^2^ = 99.04%, *p* < 0.001). The proportion estimate of *bla*_CTX-M-15_ gene from human-based studies was 47% (95% CI: 25% – 69%), for the animal studies it was 47% (95% CI: 27% – 67%) and for the environmental-based studies, it was 41% (95% CI: 33% – 50%). Between subgroups, heterogeneity was non-significant (*p* = 0.812). However, the within-group heterogeneity for both human and animal studies was significant and substantial (*I*^2^ = 99%, *p* < 0.001).

### Proportion estimates of AmpC and carbapenemase beta-lactamase genes in Nigeria

**FIGURE 4:**
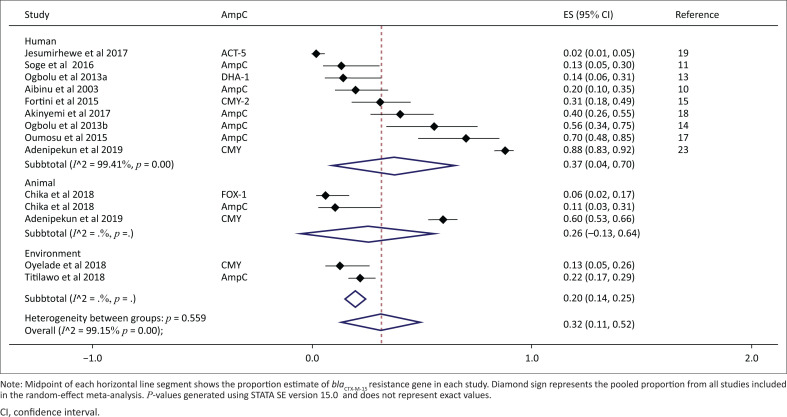
Subgroup analysis and forest plot of proportion estimates of AmpC beta-lactamases for human, animal and environmental settings in Nigeria (1990–2019).

Based on 13 studies, the overall pooled proportion of AmpC beta-lactamases was 32% (95% CI: 11% – 52%, *p* < 0.001), with the overall between-study heterogeneity significant and substantial (*I*^2^ 99.15 %, *p* < 0.001). However, the proportions of AmpC beta-lactamases reported from the studies range from 2% to 88% (Figure 4). The proportion of AmpC beta-lactamases was higher in humans at 37% (95% CI: 4% – 70%, *p* = 0.03) than in the environment at 20% (95% CI: 14% – 25%, *p* < 0.001) and animals at 26% (95% CI: 0% – 64%, *p* = 0.20).

**FIGURE 5:**
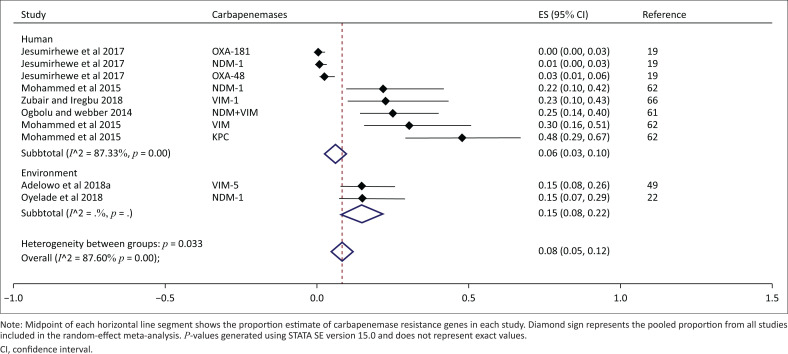
Subgroup analysis and forest plot of proportion estimates of carbapenemase beta-lactamases for human and environmental settings in Nigeria (1990–2019).

Six studies were included in the carbapenemase pooled proportion estimation (Figure 5). The overall pooled proportion of carbapenemases was 8% (95% CI: 5% – 12%, *p* < 0.001). Between the studies, the proportions of carbapenemase beta-lactamases ranged from 1% to 48%, while the overall between-study heterogeneity was significant (*I*^2^ = 87.6 %, *p* < 0.001). The proportion of carbapenemases for the environment was 15% (95% CI: 8% – 22%, *p* < 0.001) and was higher than that observed in human studies (6%; 95% CI: 3% – 10%, *p* < 0.001). Between-study heterogeneity was mostly due to studies from human setting.

## Discussion

In Africa, data on AMR are often limited due to the lack of sustainable integrated national AMR surveillance programmes. The lack of systematically collected data has impeded the proper understanding of resistance to critically important antimicrobials such as beta-lactam drugs in Africa compared to the high-income countries. This study identified the different types, distribution and proportion estimates of beta-lactamase resistance genes in Nigeria contributing to the national, continental and global molecular epidemiology of beta-lactamases. It provides data that can support ongoing efforts of integrative surveillance programmes and policies for the mitigation of AMR within Nigeria. From this review, beta-lactamases were mainly detected in the *Enterobacteriaceae* bacteria family. This higher detection in *Enterobacteriaceae* may be skewed because the studies included in this review targeted this bacterial family. Also, bacteria of the *Enterobacteriaceae* family are ubiquitous in nature, causing different infections (particularly *Escherichia coli*) in both humans and animals and can readily be maintained in the environment. Also, the emergence and occurrences of AMR due to beta-lactamases in *Enterobacteriaceae* are driven mainly by ease of acquisition of AMR genes and the rapid dissemination of resistance determinants by *Enterobacteriaceae* to other pathogenic and non-pathogenic bacteria.^2,67^

Among the 57 studies, 75% reported a double-disc synergy test for the phenotypic detection of beta-lactamases before the genotypic method. This complies with the Clinical Laboratory Standard Institute guideline for the screening and detection of beta-lactamases in bacteria. The PCR method was mostly reported; this may be due to ease of access and reduced cost compared with other advanced techniques such as WGS.^68^ While the WGS method is commonly used in high income countries for research and surveillance programmes, the use of WGS in beta-lactamase studies in Nigeria is still limited. Only two studies reported the use of WGS for the detection of beta-lactamase genes. This may be due to the limited access to the necessary equipment, expertise, and bioinformatics skill in Nigeria.

The majority of the articles published were from southern Nigeria and from human health studies. Therefore, beta-lactamases reported in this systematic review may not reflect the true geographic distribution of beta-lactamases genes in Nigeria. However, beta-lactamases were detected more in isolates from human settings, consistent with what has been reported from a similar South African review.^68^

In this review, 36 different types of beta-lactamases had been detected and reported in Nigeria. These beta-lactamases included the clinically important types such as ESBLs, AmpC, and carbapenemases that are commonly responsible for treatment failures in both human and veterinary settings.^69,70^ While previous reviews within Africa focused mainly on systematic reviews of ESBL in *Enterobacteriaceae*,^71,72,73^ this study was conducted to capture as many beta-lactamases detected in Nigeria as possible beyond ESBLs. This is because ESBLs, the AmpC-type, and the carbapenemases remain the most clinically challenging beta-lactamase resistance gene family in both human and animal health. Antibiotic resistance is recognised as a One health challenge because of the dissemination of important resistant bacteria and genes among humans, animals and the environment at a global scale.^74^ This review showed that of the 36 beta-lactamases reported in Nigeria, some of the genes detected in Nigeria were reported from more than one setting. Between human and environmental sources, five different beta-lactamases were reported while between animal and human sources, eight different genes were reported. This finding further highlights a One health AMR transmission. Also, it reveals how the environment, including food-animal production systems, could serve as reservoirs of essential AMR genes, driving transmission and colonisation as well as infection of clinically important beta-lactamase producing bacteria in humans.^6^

Epidemiologically, all the beta-lactamases detected in Nigeria have been reported from other parts of the world. Five different types of the AmpC beta-lactamase group (*bla*_ACC_, *bla*_ACT_, *bla*_CMY-2_, *bla*_DHA,_ and *bla*_FOX-1_) were reported in Nigeria to date based on this review. While *bla*_CMY-2_ is the most important AmpC-type and has the broadest geographic spread, based on this review this gene was not common in Nigeria compared to other countries such as the United States and Canada.^8^ Within Africa, there are limited reports on AmpC beta-lactamases. However, *bla*_CMY-2_ and *bla*_DHA_ have been reported in Algeria,^71^ while *bla*_ACC_, *bla*_DHA_, and *bla*_FOX__-1_ were reported in Uganda.^75^ No carbapenemase gene was reported in animals in Nigeria based on this review; however, carbapenemases have been commonly reported from wildlife, food-producing animals and companion animals from other countries.^70,76^ The lack of reports of carbapenemase from animal settings may be due to the lack of research or surveillance in this regard and not necessarily the absence of carbapenemase genes in animals from Nigeria. All the epidemiologically important carbapenemases including *bla*_KPC_, *bla*_NDM-1_, *bla*_OXA-23_, *bla*_OXA-48_, *bla*_OXA-181_, *bla*_VIM-1_, *bla*_VIM-2_ and *bla*_VIM-5_ reported in Nigeria were mostly from the human setting. These carbapenemases have been reported in many African countries including South Africa, Gabon, Angola, Senegal, Kenya, Tanzania, Morocco, Algeria, Tunisia, Libya and Egypt.^77,78^ In most cases, *bla*_ND__M-1_ and *bla*_OXA-48_ are the commonly reported carbapenemases. These carbapenemases are known to be prevalent in South Asian countries, particularly the Indian sub-continent; the trend in Africa may indicate that the global dissemination of carbapenemase-producing *Enterobacteriaceae* has reached the African continent.

Among the ESBLs, five different groups were reported including *bla*_CTX-M_, *bla*_GES,_ *bla*_OXA_, *bla*_SHV_ and *bla*_VEB_ with *bla*_CTX-M_ the most commonly reported. During the last decade, *bla*_CTX-M_–type enzymes have spread globally, becoming the most common ESBL in *Enterobacteriaceae* from both humans and animals.^79,80^ Among the different types of *bla*_CTX-M_ reported in Nigeria, *bla*_CTX-M-1_, *bla*_CTX-M-2_, *bla*_CTX-M-14_ and *bla*_CTX-M-55_ are known to be commonly detected in food animals.^4,8^ However, the internationally disseminated *bla*_CTX-M-15_ has been associated with the *E. coli* serotype O25:H4 (ST131), causing both community and hospital-acquired human infections.^79,81^ *bla*_CTX-M-15_ was also the only ESBL commonly reported in human, animal and environmental settings in this review. This revealed that *bla*_CTX-M-15_ is ubiquitous and prevalent in all environments with possible anthropozoonotic and zooanthroponotic transmissions. *bla*_CTX-M-15_ has also been commonly reported from other regions of Africa,^71^ which may suggest *bla*_CTX-M-15_ is a predominant ESBL in Africa similar to what has been reported in the United States, Europe and Asia.^82^ *bla*_SHV_ ESBLs, in particular, *bla*_SHV-12_ and *bla*_SHV-2_, reported in Nigeria have also been frequently detected in Europe and North America.^83^ However, the globally disseminated *bla*_TEM_ ESBLs, that is, *bla*_TEM-10_ and *bla*_TEM-52_, were not reported in Nigeria; narrow-spectrum *bla*_TEM-1_ coding for ampicillin resistance was common.

From the meta-analysis, subgroup analyses provide some explanation for the between-study heterogeneity and also the pooled proportion of ESBLs based on the one health distribution. The proportion estimate of ESBLs in Nigeria was 32.00% compared to 2.03% of AmpC and 8.00% of carbapenemases. This is unsurprising: even though AmpC has been found worldwide and carbapenemases are increasingly being reported, both AmpC and carbapenemases are less prevalent than ESBL globally,^5^ and this is consistent with the finding of this review. The proportion estimate of 29.00% for AmpC in the human setting in this review is slightly higher than the 28.30% estimate from Egypt,^84^ but lower than 34.00% from Canada^85^ and 39.60% from Uganda.^75^ The proportion estimate of 8.00% for carbapenemases is comparable to what has been reported from other African countries.^77^ The lower proportion of carbapenemases reported in Nigeria is encouraging considering the importance of carbapenems as last resort antimicrobials for treating cases of ESBL-producing bacterial infections. While carbapenems resistance is emerging globally at a rapid rate, surveillance and prudent use practices of carbapenems will monitor and minimise multidrug resistant bacterial infections at the national level. The proportion estimate of 32.00% for ESBL in human, animal and environmental settings from this review is higher than the 22.60% reported by a similar study from Tanzania.^73^ The proportion estimate of 35.00% for ESBLs in the human setting is comparable to proportions previously reported for different countries within Africa.^72,71^ For *bla*_CTX-M_, the proportion estimate of 34.00% for the *bla*_CTX-M_ gene in Nigeria was lower compared to 56.70% reported in Iran^86^ and 69.00% from a previous similar systematic review.^87^ However, the proportion estimate of 45.00% for *bla*_CTX-M-15_ in Nigeria was lower than 78.00% reported from both Tanzania^73^ and Sudan.^88^

None of the articles reviewed reported any risk factors associated with the occurrence of beta-lactamase resistance genes in Nigeria; however, the occurrences and proportion estimates of clinically significant beta-lactamases reported maybe due to the uncontrolled and indiscriminate use of antimicrobials as well as the lack of active infection control programmes in most animal and human settings. In Nigeria, animal and human antimicrobials can readily be purchased from both pharmaceutical and non-pharmaceutical stores without prescriptions.^89^ This is a cause for concern because indiscriminate use of antimicrobials drives resistance; also, poor hygienic practices in both community and hospital environments facilitates the spread and transmission of important multidrug resistant bacteria. In addition, extended-spectrum cephalosporins and carbapenems have been designated as critically important antimicrobials by the World Health Organization with limited alternatives in the case of treatment failure.^1^ Lastly, infections with bacteria producing ESBL or AmpC or carbapenemase may result in prolonged hospitalisation, higher treatment costs, delays in the initiation of timely and adequate antimicrobial therapy, and increased risk of morbidity and mortality.^7^ Therefore, resistance to clinically important beta-lactamases is a significant threat to public health and collaborative efforts.

### Limitations

This review is not without limitations. The literature search was limited to the PubMed^®^, Google Scholar and AJOL electronic databases; therefore, some studies may have been omitted in this review. Also, information on risk factors associated with beta-lactamase resistance was not available. This information is necessary for better explanation of the beta-lactam resistance observed in Nigeria and a better understanding of the epidemiology of beta-lactamase resistance genes in Nigeria.

### Conclusion

This review has provided information on the beta-lactamases distribution in Nigeria. Thirty-six different beta-lactamases have been reported in Nigeria with *bla*_CTX-M-15_ commonly distributed in animals, humans and the environment consistent with the reports from other African countries. Carbapenemases are most common in human settings and have not been reported in animals yet. The information provided on beta-lactamase resistance genes is necessary for better understanding of the national and global molecular epidemiology of clinically important beta-lactamase genes, especially AmpC, ESBLs and carbapenemases.
